# The Prevalence of Atypical Antipsychotics, Antidepressants, and Benzodiazepines Use in Dementia Patients in King Abdulaziz Medical City

**DOI:** 10.7759/cureus.30460

**Published:** 2022-10-19

**Authors:** Amani J Basaeed, Gada Wafia, Bashair Alhidri, Layla A Sindi, Alqassem Y Hakami, Nisreen Jastaniah

**Affiliations:** 1 Medicine, King Saud Bin Abdulaziz University for Health Sciences College of Medicine, Jeddah, SAU; 2 Family Medicine, King Saud Bin Abdulaziz University for Health Sciences College of Medicine, Jeddah, SAU; 3 Medicine and Surgery, King Saud Bin Abdulaziz University for Health Sciences College of Medicine, Jeddah, SAU; 4 Neurology, King Saud Bin Abdulaziz University for Health Sciences College of Medicine, Jeddah, SAU; 5 Geriatrics, King Abdulaziz Medical City, Ministry of National Guard - Health Affairs, Jeddah, SAU

**Keywords:** neuropsychiatric symptoms, benzodiazepines, antidepressants, atypical antipsychotics, dementia

## Abstract

Background

Dementia is a major neuropsychiatric disease defined by a progressive decline in cognitive functions. Atypical antipsychotics, antidepressants, and benzodiazepines are mainly prescribed for dementia. Many dementia pharmacological management options are associated with serious health risks. Therefore, this study aimed to determine the prevalence of antipsychotic, antidepressant, and benzodiazepine use in dementia patients in King Abdulaziz Medical City.

Methodology

A cross-sectional study was conducted at a tertiary hospital (King Abdulaziz Medical City, Jeddah) between December 2016 and January 2019. The participants were patients over the age of 65 years diagnosed with dementia. Data were collected from the medical records of the hospital after acquiring ethical approval. Patients with psychiatric diseases preceding the diagnosis of dementia, or patients with dementia-like symptoms as a side effect of any medications were excluded. The variables included were demographics, dementia subtypes, medications, and the presence or absence of chronic diseases.

Results

This study included 139 patients of whom 51.1% were males. The mean age was 82.8 ± 8.8 years. Moreover, 34.53% of the patients were prescribed medications for dementia management. Importantly, medications prescribed for dementia were classified as the following: atypical antipsychotics (20.86%), antidepressants (17.3%), and benzodiazepines (5%). The most commonly prescribed atypical antipsychotics were quetiapine (93.1%), risperidone (13.8%), and olanzapine (3.44%). For antidepressants, the most commonly prescribed medications for dementia were mirtazapine (62.5%), citalopram (45.8%), amitriptyline (8.3%), and paroxetine (4.2%). Moreover, most prescriptions for benzodiazepine were divided between lorazepam (71.4%), clonazepam (14.3%), and diazepam (14.3%).

Conclusions

This study’s results were consistent with previous epidemiological studies that have been conducted worldwide regarding the increase in the use of antipsychotics and antidepressants, with the exception of benzodiazepines. To our knowledge, there is a lack of research regarding the medications prescribed in the geriatrics age group with dementia. Therefore, the outcomes of this study recommend initiating awareness campaigns among physicians, regarding the harm of using antipsychotics, especially for this age group. Lastly, future studies should focus on increased surveillance and evaluation of drug safety warnings in dementia patients to improve the outcomes of the intervention.

## Introduction

It is expected that by 2040, 25% of Saudi Arabia’s population will be above the age of 60 [1]. As the average age of the population increases, a significant impact on morbidity, cognitive health, and physical wellness is expected to arise [2]. Prevalence of dementia, a major neuropsychiatric disease as per the Diagnostic and Statistical Manual of Mental Disorders Fifth Edition (DSM-5), which increases with age, has shown an elevation from 2-3% in the age group of 70-75 years to 20-25% among those aged 85 years old or more globally [3].

A previous study estimated that around 61% of patients with dementia experience at least one neuropsychiatric symptom (NPS) in their lifetime, including delusions and depression [4]. Many atypical antipsychotic medications, such as quetiapine and risperidone, are prescribed to alleviate NPSs in patients with dementia, such as agitation, aggression, depression, and delusions [5]. These medications are prescribed when non-pharmacological interventions have failed, or when the associated behavior causes concerns about the safety of the patients [5].

Antipsychotic medications have several side effects, including cerebrovascular events, orthostatic hypotension, thromboembolic effects, and dehydration due to excessive sedation or extrapyramidal symptoms. Among the medications used in dementia, benzodiazepines have been used to treat the side effects of NPSs, including susceptibility to falls, high dependency, and decreased cognition [6-8].

Antidepressants and mood stabilizers have shown very little efficacy for depression in dementia patients [9]. The US Food and Drug Administration, the European Medicines Agency, and the UK Medicines and Healthcare Products Regulatory Agency have issued warnings regarding the use of antipsychotics in individuals with dementia [10]. In addition, the American Geriatric Society recommended avoiding atypical antipsychotics to treat NPSs. Due to the risk of cerebrovascular adverse events, the National Institute for Health and Care Excellence (NICE) has recommended that antipsychotic drugs should not be prescribed for patients experiencing mild-to-moderate psychological and behavioral symptoms of dementia. Moreover, the prescription should be for a limited time and reviewed every three months [11].

Previous findings have documented an established risk of prescribing antipsychotic drugs to patients with dementia. Importantly, antipsychotic medications can be associated with increased risks of stroke and extrapyramidal symptoms [12,13]. Healthcare authorities in different countries have announced warnings and legislation to regulate the inappropriate prescribing of antipsychotics to dementia patients [11,14]. These findings urge medical practitioners to improve management and provide the best care for the rising elderly population. In our study, we documented the prevalence of antipsychotic, antidepressant, and benzodiazepine use in dementia patients in King Abdulaziz Medical City, as well as any association of adverse effects of these drugs in this age group.

## Materials and methods

A cross-sectional study was conducted to determine the prevalence of antipsychotic, antidepressant, and benzodiazepine use in dementia patients at the National Guard Hospital in King Abdulaziz Medical City, Jeddah, a tertiary healthcare center. The hospital offers the population of the western region different medical services and has a capacity of approximately 480 beds. Ethical approval was obtained from the Institutional Review Board (IRB) at King Abdullah International Medical Research Center (SP19/124/J). We included all male and female dementia patients above the age of 65 from December 2016 to January 2019 in the hospital. Patients who were diagnosed with any psychiatric diseases preceding the diagnosis of dementia and those with dementia-like symptoms such as confusion and urinary incontinence side effects of taking antipsychotic medications were excluded.

The sample size was calculated as 139 participants using a Raosoft calculator with a 95% confidence interval, 5% margin of error, and 32% prevalence rate according to a previous report [15]. A consecutive sampling technique was used to select the participants in this study, and the electronic files were reviewed to extract relevant data. All patients’ personal data including name, contact details, and diagnosis remained confidential.

The data were collected from the Best-Care system in the National Guard Hospital, Jeddah, using a standardized data collection sheet. The sheet had two main sections, namely, demographics and prescription profile. The demographics included serial coding number, gender, age, diagnosis, and presence or absence of comorbidities, such as diabetes, hypertension, cardiovascular diseases, and other psychiatric diseases. In the prescription section, the class of medication was documented (atypical antipsychotics, antidepressants, and benzodiazepines), along with their sub-classification.

Data analysis was done using SPSS version 22 (IBM Corp., Armonk, NY, USA). For qualitative data, simple descriptive statistics were used (e.g., numbers, and percentages for categorical and nominal variables). For quantitative data, the mean and standard deviation were used. For the comparative analysis, we used the chi-square test and Fisher exact test. The level of significance was considered at a p-value of ≤0.05.

## Results

In this study, 139 patients were included, and out of those 71 (51.1%) were male. The mean age of the participants was 82.8 ± 8.8 years. Participants identified with specific comorbidities were as follows: diabetes (89, 64%), hypertension (114, 82%), cardiovascular diseases (73, 52.5%), and psychiatric diseases other than dementia (13, 9.4%) (Table 1).

**Table 1:** Characteristics and comorbidities of the studied population (N = 139). SD: standard deviation

Variable	N (%)
Age	
Mean ± SD	82.8 ± 8.8
Gender	
Male	71 (51.1)
Female	68 (48.9)
Comorbidities	
Diabetes	89 (64)
Hypertension	114 (82)
Cardiovascular diseases	73 (52.2)
Other psychiatric diseases	13 (9.4)

**Figure 1:**
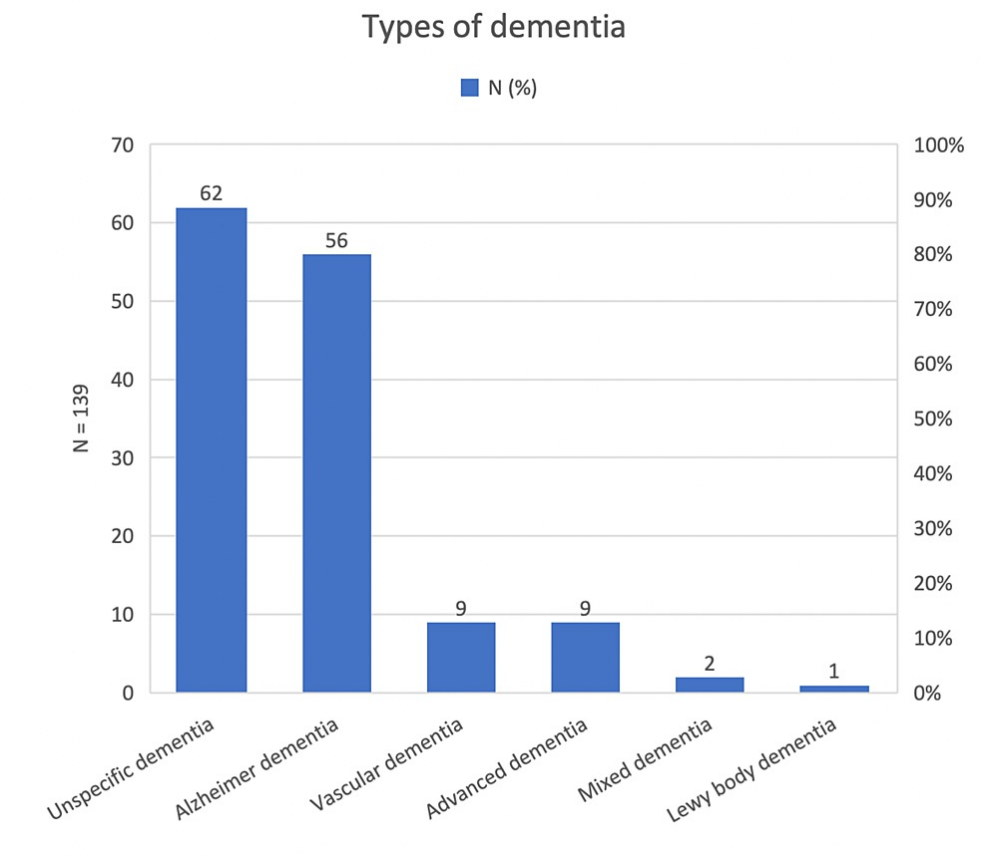
Types of dementia among the studied population (N = 139).

The types of dementia noted in the participants included Alzheimer’s dementia (56, 40.3%), vascular dementia (9, 6.5%), advanced dementia (9, 6.5), mixed dementia (2, 1.4%), Lewy body dementia (1, 0.7%), and unspecified dementia (62, 44.6%) (Figure 1). Regarding the prescription section, 48 (34.53%) participants had prescriptions for dementia. The use of atypical antipsychotics was found to be the highest (29, 20.86%), followed by antidepressants (24, 17.3%), and, finally, benzodiazepines (7, 5%) (Table 2).

**Table 2:** Frequencies and percentages of psychotropic medications among the studied population (N = 139).

Class	N(%)
Atypical antipsychotics	N = 34 (24.5%)
Quetiapine	27 (79.4%)
Risperidone	4 (11.8%)
Olanzapine	1 (2.9%)
Antidepressants	N = 24 (17.3%)
Mirtazapine	15 (62.5%)
Citalopram	11 (45.8%)
Amitriptyline	2 (8.3%)
Paroxetine	1 (4.2%)
Benzodiazepines	N = 7 (5%)
Lorazepam	5 (71.4%)
Clonazepam	1 (14.3%)
Diazepam	1 (14.3%)

Our data showed that the highest subclassification of atypical antipsychotics used was quetiapine (27, 93.10%), followed by risperidone (4, 13.8%), and olanzapine (1, 3.44%) (Table 2). Antidepressants were subclassified as mirtazapine (15, 62.5%), citalopram (11, 45.8%), amitriptyline (2, 8.3%), and paroxetine (1, 4.2%) (Table 2). Lastly, lorazepam usage consisted of 71.4% of all benzodiazepines (5, 71.4%), followed by clonazepam (1, 14.3%), and diazepam (1, 14.3%) (Table 2), with some patients being on combined therapy. The statistical analysis revealed no significant difference between gender and the prescribed medications (Table 3).

**Table 3:** Correlation between medications’ classification among genders. P-value < 0.05 was statistically significant.

Class	Gender		P-value
	Male	Female	
Atypical antipsychotics	19	15	0.519
Quetiapine	17	10	0.169
Risperidone	2	2	1.000
Olanzapine	1	2	1.000
Antidepressants	13	11	0.739
Mirtazapine	7	8	0.717
Citalopram	6	5	0.811
Amitriptyline	1	1	1.000
Paroxetine	0	1	0.489
Benzodiazepines	4	3	1.000
Lorazepam	3	2	1.000
Clonazepam	0	2	0.238
Diazepam	1	0	1.000

## Discussion

The rates of prescription in this cross-sectional study confirm that among the three medication classes studied, atypical antipsychotic medications were the most prescribed for the management of elderly dementia patients at the National Guard Hospital in Jeddah, followed by antidepressants, and, lastly, benzodiazepines. Quetiapine was found to be increasingly ordered among the atypical antipsychotic medication class. Mirtazapine and citalopram were the most prescribed antidepressants, while the prescription rate of lorazepam was the highest among benzodiazepines.

High prevalence of atypical antipsychotics

The major concern is the high prevalence of the prescription of atypical antipsychotics among the studied population, which constitutes 20.86% of the patients (N = 29). Multiple studies have found a similar higher rate of prescribing atypical antipsychotics compared to other medication classes. A study conducted in the United States on the prevalence of prescription of antipsychotics in dementia patients in nursing homes revealed that the rate was around 32.88%, with the use of atypical antipsychotics being significantly higher (31.63%) than typical antipsychotics (1.75%) [15]. Another study conducted in European long-term care facilities revealed an overall prevalence of antipsychotic use to be at 32.8%, concluding that seven in ten dementia patients used atypical antipsychotics [16].

In Taiwan, the prevalence of antipsychotic medication utilization was also high, reaching 26.5%. The study attributed the increased prescription rates over the past nine years to the replacement of typical antipsychotics with atypical medications [17]. Atypical antipsychotic use for dementia patients was the highest despite it being considered off-label use for dementia treatment [5]. This increased prescription might be due to the significant improvements in agitation occurring in dementia patients [18], which is the principal implication for prescribing antipsychotic medication in this group [19].

Our findings suggest that patients in the studied population were more likely to have been prescribed quetiapine (27, 93.10%). A systemic review that included 12 meta-analyses revealed that quetiapine has a favorable anesthetic outcome and fewer extrapyramidal side effects compared to other atypical antipsychotics [18]. There is solid evidence from a large population study that the number needed to harm (NNH), which is a measure of how many people need to be treated for one person to have a particular adverse effect, for quetiapine is 50 (95% CI = 30-150). Thus, quetiapine was the least medication for NNH among other atypical antipsychotics and antidepressants compared to non-users [20]. This finding supports the preference for prescribing quetiapine among other medications in the class of atypical antipsychotics.

Prevalence of antidepressants

Considering this study, antidepressants were the second most common class of prescribed medication (24, 17.3%). Both mirtazapine (15, 62.5%) and citalopram (11, 45.8%) were the most used in this class. In dementia patients, antidepressants are mainly prescribed for agitation symptoms [21]. However, the usage of antidepressants is highly associated with undesirable side effects, such as recurrent falls, fractures, and increased mortality rates [22]. According to a study conducted at Michigan university, antidepressants were correlated with a low yet significant increase in mortality [20].

Prevalence of benzodiazepines

In our study, the prescription of benzodiazepines as management for behavioral and psychological symptoms associated with dementia (BPSD) was found to be the least compared to other medication classes (7, 5%). Benzodiazepines are associated with increased side effects, such as sedation, increased risk of falls, diminished cognition, and the risk of dependency [8]. Therefore, the use of benzodiazepines should be limited to certain groups with a restricted duration of treatment and prescribed with caution [8].

A study in Sweden with a sample size of 3,395 dementia participants revealed that antipsychotic use was higher among men, and antidepressants were the preferable choice among women. However, no gender difference was reported in benzodiazepine use [23]. This higher use of antipsychotics among men might be attributed to the increased reporting of aggressive symptoms. While women with dementia showed more depressive symptoms, antidepressants were the preferable choice [23]. On the contrary, our study showed no significant difference between genders regarding the medication classes. The difference in the results might be due to the larger sample size in the previous study.

One limitation of this study is that the data collected were restricted to patients in the National Guard Hospital, Jeddah. Further multicenter investigations should include a more representative sample size. Additionally, the diagnosis of dementia was not mentioned clearly as a primary diagnosis in patients’ files.

## Conclusions

The prevalence of dementia has increased globally, as previous studies have demonstrated, and with this high prevalence, a larger number of medications are prescribed to ease NPSs of dementia patients, including the use of antipsychotics, antidepressants, and benzodiazepines. Overall, the findings of this research were consistent with previous worldwide epidemiological studies regarding the increased utilization of antipsychotics and antidepressants but not benzodiazepines for the treatment of NPSs in dementia patients.

The findings of our study demonstrating an increased antipsychotic prescription rate suggest the need for more effort to raise awareness regarding the use of atypical antipsychotic medications in the management of dementia. The numerous side effects associated with pharmacological interventions to manage symptoms of BPSD urge physicians to implement non-pharmacological approaches as a first-line option in the management of dementia.

More broadly, additional studies are needed to report the potential side effects of using antipsychotics, antidepressants, and benzodiazepines among dementia patients, as well as drug interactions with other comorbidities. Future studies should focus on evaluating the quality of life with medication use in dementia patients and compare it with non-pharmacological approaches.
